# Phytochemical Analysis and Antifungal Potentiating Activity of Extracts from Loquat (*Eriobotrya japonica*) against *Cryptococcus neoformans* Clinical Isolates

**DOI:** 10.1155/2022/6626834

**Published:** 2022-04-14

**Authors:** Borel Ndezo Bisso, Prudence Ngalula Kayoka-Kabongo, Roland Tchuenteu Tchuenguem, Jean Paul Dzoyem

**Affiliations:** ^1^Department of Biochemistry, Faculty of Science, University of Dschang, Dschang, Cameroon; ^2^Department of Agriculture and Animal Health, College of Agriculture and Environmental Sciences, University of South Africa, Florida, South Africa

## Abstract

*Eriobotrya japonica* (loquat) has been used in African traditional medicine with numerous beneficial health effects. The extracts from loquat contain several bioactive compounds with a plethora of pharmacological properties. However, a scientific study on the activity against the aetiological agent of cryptococcosis has not yet been reported. Therefore, this study aimed to investigate the antifungal potential of various extracts from *Eriobotrya japonica* against clinical isolates of *Cryptococcus neoformans*. Quantitative and qualitative phytochemical analyses of extracts were made by following standard procedures. The broth microdilution method and the checkerboard methods were used to determine the antifungal activity and the combination of extracts with antifungals drugs. The methanol extract of seeds and the hexane extract of leaves exhibited the best significant antifungal activity with MIC values of 32 *µ*g/mL. Furthermore, the combination of both extracts with nystatin and clotrimazole showed synergistic interactions with a 32-fold reduction in the MIC values of nystatin. Our findings indicate that *Eriobotrya japonica* extracts are a potential source of new antifungals that could be developed for use in the treatment of cryptococcosis. The anticryptococcal and antifungal activities potentiating activity of the studied extracts indicate their potential in the management of cryptococcosis. Further study should be considered to identify the bioactive principles against *Cryptococcus neoformans*.

## 1. Introduction

Cryptococcosis is a fungal infection, caused by encapsulated opportunistic yeast in the genus *Cryptococcus* [1]. This yeast is gaining prominence as a pathogen capable of widespread disease outbreaks in vulnerable populations, causing life-threatening meningoencephalitis mostly in patients with human immunodeficiency virus (HIV) [2]. The global incidence of cryptococcosis is estimated to kill over 180,000 people per year, with 75% of deaths occurring in sub-Saharan Africa, and the mortality of cryptococcal meningitis in low-income countries may exceed 70% [3]. Despite substantial improvement in the management of clinical events like AIDS, the numbers of cases of cryptococcosis remain very high [4–6]. Although several antifungal agents such as amphotericin B, azoles, and 5-flucytosine are available for treatment, side effects associated with amphotericin B and antifungal drug resistance hamper the efficacy of these antifungals. Additionally, morbidity and mortality rates of cryptococcosis remain high with this fungal infection [7, 8]. Therefore, it is important to search for more potent new antifungal agents with fewer side effects. A potential promising source of new antifungal agents is medicinal plants since they have been used by local populations to treat various infections for a long time, which has directed several pharmacological studies [9]. Medicinal plants and their phytochemicals constituents have been used for their effective antimicrobial effects from ancient times, and there is an increasing trend for the development of plant-based natural products for the prevention and treatment of pathogenic diseases [10]. Several studies have reported the antifungal efficacy of different medicinal plant extracts [11]. In addition, a new alternative approach is the use of combination therapy between the available antifungal agents and medicinal plant extracts, and this strategy has shown promising results [12]. Some studies highlighted the use of plant natural products and plant extracts in combinations with antibiotics as having particular promise for rapidly developing new and effective agents to combat pathogens resistant to conventional antibiotic therapies [13]. *Eriobotrya japonica* (Thunb.) Lindl, belonging to the Rosaceae family, is a subtropical tree traditionally used as medicine for the treatment of many diseases including microbial infections such as cough, chronic bronchitis, and skin infections [14]. Previous studies have reported the antimicrobial, antioxidant, anti-inflammatory, antidiabetic, and anticancer activities of *E. japonica* extracts [14, 15]. The antifungal effect of loquat leaf extract on postharvest citrus fruits against fungal pathogens showed significant inhibitory effects [16]. However, there is no study reporting the evaluation of its properties against the aetiological agent of cryptococcosis or its ability to potentiate the activity of existing antifungals. Therefore, the present study aims to evaluate the antifungal activity of *E. japonica* extracts and their synergistic effect in association with nystatin and clotrimazole against clinical isolates of *Cryptococcus neoformans*.

## 2. Materials and Methods

### 2.1. Plant Material and Extraction

The leaves, seeds, and bark of *Eriobotrya japonica* (Thunb.) Lindl at Balatchi in the west region of Cameroon in October 2019 were collected. The plant materials were identified at the Cameroon National Herbarium (Yaoundé), where a voucher specimen was deposited under the reference number: 44164/HCN. The parts of the plant were dried at room temperature in the shade and then were powdered. Then, 100 g powder of each part of the plant was macerated with 500 mL of each solvent (hexane, chloroform, ethyl acetate, and methanol) for 48 h at room temperature. The extracts were filtered using Whatman no.1 filter paper, and solvents were evaporated using a rotary evaporator. The extracts were stored at 4°C until further use. The extraction yield (%) was calculated as follows:

Extraction yield (%) = (weight of the extract after evaporating solvent and drying/dry weight of the sample) *x* 100.

### 2.2. Phytochemical Screening

Qualitative phytochemical screening extracts were carried out by standard chemical methods as described by Harbone (1984) [17]. Total phenolic content (TPC) and total flavonoids content (TFC) were determined spectrophotometrically using Folin–Ciocalteu assay as previously reported [18].

### 2.3. Antifungal Assay

#### 2.3.1. Microorganisms, Culture Media, and Antifungals

The microorganisms used in this work included nine clinical isolates of *Cryptococcus neoformans*. These isolates were identified by serotyping by multiplex PCR in a previous study [19]. Sabouraud dextrose agar and broth were used, respectively, for the activation of yeasts and the determination of minimum inhibitory concentration (MIC), minimum fungicidal concentration (MFC), and fractional inhibitory concentration (FIC). Nystatin and clotrimazole purchased from Sigma-Aldrich Chemie (Steinheim, Germany) were used as reference antifungals.

### 2.4. MIC and MFC Determination

The determination of MIC and MFC of the extracts and antifungals (nystatin and clotrimazole) against the nine isolates of *Cryptococcus neoformans* was performed by the broth microdilution method as previously described [20].

#### 2.4.1. Determination of Fractional Inhibitory Concentration (FIC) and Fractional Inhibitory Concentration Index (FICI)

After MICs were determined, the two most active extracts were selected to evaluate their effect in association with nystatin and clotrimazole. The checkerboard method used was the broth microdilution assay, miniaturized in a 96-well plate as described by Kong et al. (2020) with slight modifications [21]. Briefly, 50 *µ*L of culture media were introduced in each well of the two microplates. In the first microplate, 50 *µ*L of the extract was introduced in each well of line H and two-fold dilution was done from line H to line B. In the second microplate, 50 *µ*L of antifungal was introduced in each well of column 12 except in well A12. Then, a two-fold dilution was made from column 12 to column 2. After dilution, the contents of the wells of the second microplate were transferred to the corresponding wells in the first microplate. Then, 100 *µ*L of inoculums at 1.5 × 104 CFU/mL was added to each well of the microplate except in well A12, and the microplate was incubated aerobically at 35°C for 48 hours. Well A1 containing yeast and SDB was used as positive growth control, while well A12 without yeast was used as negative growth control. Each experiment was performed thrice in duplicate. For each experiment, the MIC value was defined as the lowest concentration of extracts or antifungals for which no visual growth was observed. FICIs were calculated as follows: FICI = FICext + FICatf, where FICext is the MIC of extract in the combination/MIC of extract alone, and FICatf is the MIC of antifungal in the combination/MIC of antifungal alone. The interaction was classified as synergistic when FICI values < 0.5, indifferent when 0.5 ≤  FICI ≤4, and antagonistic when FICI  > 4.

## 3. Results and Discussion

Using hexane, ethyl acetate, chloroform, and methanol as extraction solvents, twelve different extracts were prepared from seeds, leaves, and bark of *Eriobotrya japonica*. Qualitative phytochemical studies of the obtained extracts showed that all the solvents used extracted almost similar classes of phytochemicals (Table 1). Similarly, only minor differences in phytochemical profiles were noted between the three parts of the plant collected. Unlike phenols, polyphenols, flavonoids, and triterpenoids that were present in all the extracts, alkaloids were found only in the ethyl acetate extract of leaves and hexane extract of bark, while saponins were found only in the methanol extract of leaves and the methanol extract of bark. The observed diversity of extracts in secondary metabolites can be explained by the difference in polarity of the extraction solvents used or by the part of the plant used. A broad range of important phytochemicals had been detected in loquat, like phenols, alkaloids, cardiac glycosides, flavonoids, mucilage, gums, and phytosterol [22]. The results of extraction yield (Table 1) showed that the methanol with the highest polarity gave the highest yield of 8.5%, 6.68%, and 5.94%, respectively, in the seeds, leaves, and bark. The lowest yields 0.38%, 0.53%, and 0.79% were obtained with the chloroform extract of seeds, the ethyl acetate extract of seeds, and the chloroform extract of bark, respectively. These findings indicate that methanol is the best solvent for extraction, while the leaves are the plant part containing the highest amount of secondary metabolites. In the same view, other studies reported the highest extraction yield from the methanol extract of the leaves of *Eriobotrya japonica* [23].

**Figure 1:**
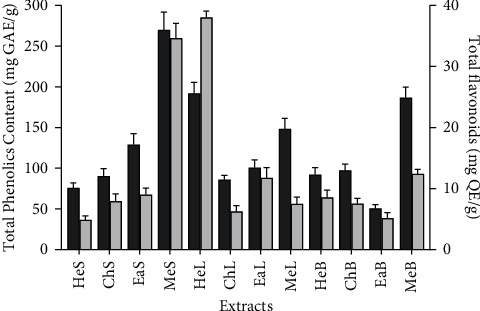
Total phenolics and flavonoids contents of extracts of *E. japonica*. HeS, hexane extract of seeds; ChS, chloroform extract of seeds; EaS, ethyl acetate extract of seeds; MeS, methanol extract of seeds; HeL, hexane extract of leaves; ChL, chloroform extract of leaves; EaL, ethyl acetate extract of leaves; MeL, methanol extract of leaves; HeB, hexane extract of bark; ChB, chloroform extract of bark; EaB, ethyl acetate extract of bark; MeB, methanol extract of bark.

Quantitative phytochemical analysis was also performed to determine the phenolic content and flavonoids content of extracts. The results shown in Figure 1 show that the highest amount of phenolics compounds was present in the methanol extract of seeds (MeS) (270.07 mg GAE/g) and the hexane extract of leaves (HeL) (191.98 mg GAE/g). Consistent with TPC, the TFC of MeS and HeL extracts was higher than that of all other extracts (34.62 mg QE/g and 38.03 mg QE/g for MeS and HeL, respectively). This trend was not similar to that observed for the extraction yield, providing evidence that phenolics compounds are not necessary, the major secondary metabolites extracted. Studies revealed values of 0.3822 mg QAE/mg and 3.810 mg GAE/mg, respectively, for methanolic and hexane extracts of *Eriobotrya japonica* [23]. An elevated amount of total flavonoids and total phenolic content have also been reported from the methanol extract of loquat bark [24].

The growth-inhibiting effect of *Eriobotrya japonica* extracts assessed against nine *Cryptococcus neoformans* isolates as determined by MICs and MFCs is given in Table 2. The antifungal activity was considered as significant when the MIC was less than 100 *μ*g/mL, moderate when the MIC was between 100 and 625 *μ*g/mL, and low when the MIC was greater than 625 *μ*g/mL [25]. Therefore, MeS and HeL extracts revealed significant antifungal activity with MIC values of 32 *μ*g/mL, followed by EaS and HeS extracts with MIC values of 64 *μ*g/mL each. All the extracts showed moderate activity against at least one of the nine isolates tested. The lowest MFC (128 *µ*g/mL) was obtained with MeS and HeL extracts.

This is the first study reporting the antifungal activity of *Eriobotrya japonica* extracts against *Cryptococcus neoformans*. Rashed et al. (2014) reported that methanol extract (80%) of stems of loquat (by maceration) demonstrated the antimicrobial effect versus strains of fungi and bacteria related to the presence of triterpenes and flavonoids [26]. This observation could be confirmed in our study since the most actives extracts (MeS and HeL) were those with the highest amount of total flavonoid and total phenolic content. Therefore, the antifungal activity observed could be related to the high total flavonoids and total phenolic content of extracts. The antimicrobial activity of phenolic compounds has been extensively documented [27, 28]. Furthermore, the potential use of phenolic compounds as antifungal agents has been reported [29].

MeS and HeL appear as the most actives extracts and were therefore selected for the combination study with nystatin and clotrimazole. The results of the combination interactions are given in Table 3. There was a decrease in the MICs values of both nystatin and clotrimazole when combined with extracts. The overall FICI values ranged between 0.04 and 8.06 which led to 24 synergistic interactions of synergies, 6 indifferences, and 6 antagonistic interactions. The MIC values of nystatin in combination ranged from 1 to 8 *μ*g/mL with a maximum MIC reduction of 32-fold. The combination of MeS with nystatin gave a synergistic effect against all the tested isolates, but when combined with clotrimazole, two indifferences and two antagonistic interactions were observed. Only one indifference and one antagonistic interaction were observed when HeL was associated with nystatin. The synergistic effect observed in MeS and HeL extracts could still be related to their high content in phenolic compounds. Phenolic compounds have been shown to exert synergistic activity against different fungal strains [30]. The combination of carvacrol with the nystatin indicated synergistic interactions, while there was no antagonistic interaction [31].

## 4. Conclusion

The results of this present study suggest that the methanol extract of the seeds of *Eriobotrya japonica* with nystatin is the best combination that effectively inhibits the growth of *Cryptococcus neoformans* in vitro and synergism was demonstrated for all the isolates used. Therefore, this extract is a potential source of new antifungals that could be developed for use in the treatment of cryptococcosis. Further study should be considered to identify the bioactive principles against *Cryptococcus neoformans*.

## Figures and Tables

**Table 1:** Phytochemical screening of leaf, seed, and bark extracts of *E. japonica*.

Extracts	Extraction yield (%)	Phytoconstituents									
		Alk	Athc	Athq	Flav	Phe	Pphe	Sap	Tan	Std	Tri
HeS	2.84	−	−	−	+	+	+	−	+	+	+
ChS	0.38	−	−	−	+	+	+	−	+	+	+
EaS	0.53	−	+	+	+	+	+	−	−	−	+
MeS	6.68	−	+	−	+	+	+	−	+	−	+
HeL	4.52	−	−	−	+	+	+	−	+	+	+
ChL	2.28	−	−	−	+	+	+	−	+	+	+
EaL	1.79	+	−	−	+	+	+	−	+	+	+
MeL	8.5	−	+	−	+	+	+	+	+	−	+
HeB	4.41	+	−	−	+	+	+	−	+	+	+
ChB	0.79	−	−	−	+	+	+	−	+	+	+
EaB	2.31	−	+	+	+	+	+	−	−	+	+
MeB	5.94	−	+	+	+	+	+	+	−	−	+

+, presence; −, absence; Alk, alkaloids; Athc, anthocyanins; Athq, anthraquinones; Flav, flavonoids; Phe, phenols; Pphe, polyphenols; Sap, saponins; Tan, tannins; Std, steroids; Tri, triterpenoids; HeS, hexane extract of seeds; ChS, chloroform extract of seeds; EaS, ethyl acetate extract of seeds; MeS, methanol extract of seeds; HeL, hexane extract of leaves; ChL, chloroform extract of leaves; EaL, ethyl acetate extract of leaves; MeL, methanol extract of leaves; HeB, hexane extract of bark; ChB, chloroform extract of bark; EaB, ethyl acetate extract of bark; MeB, methanol extract of bark.

**Table 2:** Antifungal activity of *E. japonica* extracts and antifungals against clinical isolates of *C. neoformans*.

Extracts	Isolates																			
	CN		CN169		CN173		CN047		CN091		CN165		CN118		CN046		CN096		CN158	
	MIC	MFC	MIC	MFC	MIC	MFC	MIC	MFC	MIC	MFC	MIC	MFC	MIC	MFC	MIC	MFC	MIC	MFC	MIC	MFC
HeS	512	-	1024	-	512	-	1024	-	256	-	512	-	**64**	256	1024	-	256	-	512	-
ChS	1024	-	1024	-	1024	-	512	-	512	-	1024	-	512	-	512	-	1024	-	1024	-
EaS	1024	-	512	-	1024	-	**64**	256	1024	-	1024	-	256	-	256	1024	512	-	512	-
MeS	1024	-	512	-	512	-	**32**	128	512	-	256	1024	512	-	128	512	128	1024	512	-
HeL	1024	-	512	-	1024	-	512	-	256	512	**32**	256	**32**	128	**32**	128	256	512	128	256
ChL	256	-	512	-	1024	-	1024	-	1024	-	512	-	256	512	1024	-	512	-	1024	-
EaL	512	-	1024	-	1024	-	1024	-	512	-	1024	-	1024	-	512	-	1024	-	512	-
MeL	512	-	512	-	1024	-	256	-	1024	-	512	-	1024	-	256	-	512	-	512	-
HeB	1024	-	1024	-	1024	-	512	-	512	-	512	-	1024	-	512	-	1024	-	1024	-
ChB	1024	-	512	-	1024	-	1024	-	512	-	512	-	512	-	1024	-	512	-	1024	-
EaB	512	-	256	1024	256	512	256	1024	128	512	1024	-	1024	-	512	-	512	-	1024	-
MeB	512	-	128	512	1024	-	1024	-	256	1024	1024	-	512	-	256	512	128	512	512	-
Nystatin	8	8	32	256	16	32	4	8	8	128	16	64	4	8	32	128	4	8	4	8
Clotrimazole	16	64	32	128	2	8	2	16	2	16	8	32	4	32	4	32	4	16	2	16

−, >1024 *µ*g/mL for extracts; HeS, hexane extract of seed; ChS, chloroform extract of seed; EaS, ethyl acetate extract of seed; MeS, methanol extract of seed; HeL, hexane extract of leaves; ChL, chloroform extract of leaves; EaL, ethyl acetate extract of leaves; MeL, methanol extract of leaves; HeB, hexane extract of bark; ChB, chloroform extract of bark; EaB, ethyl acetate extract of bark; MeB, methanol extract of bark. Bold values represent significant antifungal activity.

**Table 3:** Effect of the combination of methanol extract of seeds (MeS) and hexane extract of leaves (HeL) with nystatin and clotrimazole.

	MeS + nystatin		MeS + clotrimazole		HeL + nystatin		HeL + clotrimazole	
	MIC reduction fold	FICI (interpretation)	MIC reduction fold	FICI (interpretation)	MIC reduction fold	FICI (interpretation)	MIC reduction fold	FICI (interpretation)
CN	8	0.13 (Syn)	4	0.25 (Syn)	8	0.13 (Syn)	4	0.25 (Syn)
CN169	4	0.27 (Syn)	32	0.03 (Syn)	32	0.03 (Syn)	4	0.27 (Syn)
CN173	16	0.06 (Syn)	0.5	2.01 (Ind)	16	0.06 (Syn)	2	0.50 (Ind)
CN047	4	0.28 (Syn)	2	0.53 (Syn)	4	0.25 (Syn)	0.25	4.02 (Ant)
CN091	2	0.25 (Syn)	0.5	4.02 (Ant)	4	0.25 (Syn)	0.125	8.06 (Ant)
CN165	16	0.07 (Syn)	8	0.13 (Syn)	0.25	6.00 (Ant)	4	0.31 (Syn)
CN118	4	0.25 (Syn)	4	0.25 (Syn)	4	0.28 (Syn)	0.25	4.50 (Ant)
CN46	32	0.04 (Syn)	0.5	2.06 (Ind)	4	0.50 (Ind)	0.5	2.25 (Ind)
CN96	4	0.26 (Syn)	0.25	4.13 (Ant)	4	0.25 (Syn)	2	0.51 (Ind)

Syn, synergistic; Ind, indifferent; Ant, antagonistic.

## Data Availability

The data used to support the findings of this study are available from the corresponding author upon request.
